# A decreased risk of meningioma in women smokers was only observed in American studies rather than studies conducted in other countries: a systematic review and meta-analysis

**DOI:** 10.1186/s41016-021-00261-1

**Published:** 2021-11-01

**Authors:** Ping Zhong, Yiting Lin, Ting Chen

**Affiliations:** 1 BE and Phase I Clinical Trial Center, The First Affiliated Hospital of Xiamen University, Xiamen, People’s Republic of China; 2 Department of Respiratory and Critical Care Medicine, Xiamen Haicang Hospital, Xiamen, People’s Republic of China; 3 Department of Medical Examination and Blood Collection, Xiamen Blood Center, Xiamen, People’s Republic of China

**Keywords:** Smoking, Meningioma, Risk factor, Meta-analysis

## Abstract

**Background:**

Whether smoking is related to a decreased risk of meningioma in women is still controversial. We conducted a systematic review and meta-analysis examining the association between smoking and risk of meningiomas in women.

**Methods:**

Two authors independently performed a systematic literature review in the PubMed, Cochrane Library, and EMBASE databases. We identified case-control and cohort studies quantifying associations between smoking and risk of meningioma in women. A meta-analysis by pooling studies was performed according to the multivariate-adjusted risk estimates and 95% confidence intervals (CIs) preferentially. We further conducted additional subgroup and sensitivity analyses to explore possible explanations of the results.

**Results:**

A total of seven observational studies were included, with a total of 2132 female patients diagnosed with meningiomas. Ever smoking was associated with a significantly reduced risk of meningioma in women, with pooled odds ratio (OR) of 0.83 (95% CI 0.70–0.98). Similar findings were noted for current (OR 0.78, 95% CI 0.66–0.93) and past (OR 0.82, 95% CI 0.71–0.94) smokers. However, considering the areas, the OR of ever smoking was 0.77 (95% CI 0.68–0.87) in three American studies, but 0.99 (95% CI 0.73–1.35) in four studies conducted in other countries.

**Conclusions:**

Based on limited epidemiological evidence, a decreased risk of meningioma in women smokers was only observed in American studies rather than studies conducted in other countries.

## Background

Meningiomas are suggested to be among the most common primitive tumors of the central nervous system and account for about a third of all intracranial tumors [1–3]. Although the majority of meningiomas are benign, the overall 10 years survival is still less than 85% and the 5-year rate of tumor recurrence is about 20%, even for completely removed patients [3]. Women are twice as likely as men to develop meningioma, and the incidence of operated meningiomas is three times more frequent in women than men [2–4]. However, the etiology of meningiomas remains largely unexplored. Intrinsic factors (e.g., sex, atopy, ethnic, genetic polymorphisms), environmental factors (e.g., electromagnetic radiation, nutrition, pesticides, hormonal factors, occupation), and other factors (e.g. smoking, drinking, and head trauma) are suggested to be possible risk factors for the development of meningiomas [1, 2, 5–7].

It is now well established from a variety of studies, that smoking is associated with a wide range of tumors, including lung cancer, liver cancer, ovarian cancer, prostate cancer, and others [8–12]. To date, several attempts have been made to explore the possible relationship between smoking and meningiomas [13–19]. But these results are still inconsistent. As meningiomas develop more frequently in women than in men, the etiology of meningiomas in women has received considerable critical attention. Prior observational studies have shown that smoking tends to reduce the risk of meningioma in women, but most of them appear to be not statistically significant [13–17]. Meanwhile, a previous meta-analysis indicated that no significant risk existed in the relationship of smoking and meningioma in women [20]. However, a population-based study and its incidental meta-analysis demonstrated that a significant negative association has existed between the two [17]. After that, another updated study found no evidence of an association between smoking and meningioma in women [21].

It is interesting to note that women smokers in American studies tended to had a significantly decreased risk of meningioma [13 17], whereas women smokers in studies conducted in other countries showed no significantly decreased risk of meningioma [14 15 21]. Therefore, whether the association between smoking and risk of meningioma in women differs across countries is still poorly understood. To the best of our knowledge, no formal systematic review and meta-analysis based on the existing epidemiological data is available regarding the association between smoking and meningioma in women in different countries. To address this research gap, based on the updated epidemiological data, we performed a formal systematic review and meta-analysis of smoking in relation to risk of meningioma in women, with a special focus on the difference of relationship in different countries.

## Methods

### Literature search

The meta-analysis was carried out following the guidelines from the Meta-analysis of Observational Studies in Epidemiology Group [22]. In December 2020, two authors (YL and TC) independently performed a systematic literature review in the PubMed, Cochrane Library, and EMBASE databases. The following search terms were used: (1) smoking, smoke, tobacco, and cigarette; (2) meningeal neoplasms, meningeal tumor, meningioma, brain tumor, and central nervous system tumor. The same authors retrieved and independently assessed potentially relevant studies reporting information on the association between smoking and meningioma, and checked the reference list of all articles of interest to retrieve other pertinent papers. Meanwhile, all the original publications included in the meta-analysis, pooled analysis, and systematic reviews were assessed as well. However, unpublished studies and abstracts were not included. No researches were excluded a priori for the weakness of study design or data quality. Non-English reports, unpublished studies, conference proceedings, dissertations, and these publications were not considered. The full text of these potentially eligible studies was retrieved and independently assessed for eligibility by the two reviewers. Discrepancies between the two reviewers were discussed and solved. In case of disagreement, a specialist helped to find a final decision.

### Inclusion and exclusion criteria

Two literature reviewers (PZ and TC) evaluated studies independently for possible inclusion and resolved any discrepancies by discussion. The following inclusion criteria were applied: (1) used a cohort or case-control study design; (2) availability of a quantitative estimate of the associations between smoking and risk of meningiomas; (3) provided the relative risk (RR) or odds ratio (OR) with confidence intervals (CIs); (4) availability of at least one of the following smoking exposure variables: never versus ever, past, current, or passive smoking; (5) could get raw data of female population; (6) when multiple reports were based on the same target population, the most informative one was included in this meta-analysis.

### Data extraction

Titles and/or abstracts of reviews were screened independently by two reviewers to identify publications that potentially meet the inclusion criteria outlined above. No study will be excluded a priori for the weakness of design or data quality. The full texts of all the retained original articles were retrieved. A standardized form was used to extract data from each identified publication. Relevant information included: study name; authors; country; period of publication; gender; sample sizes (cases, controls or non-cases or cohort size); study design; variables of adjustment; availability of data on smoking; risk estimates (RRs or ORs) and their corresponding 95% CIs.

### Statistical analysis

All statistical analyses were used by STATA 12 (StataCorp, College Station, TX, USA). The multivariate-adjusted risk estimates and 95% CIs were used to estimate the pooled risk of smoking in meningioma development. For case-control studies, the OR was used as estimates of the RR because meningioma is sufficiently rare. When cohort studies reported only crude data and no information on person-years, we treated it as a control study using noncases as controls. The potential heterogeneity between publications was assessed by *X*^2^-based *Q* statistical test and the *I*^2^ test. Heterogeneity was considered significant when *I*^2^ > 50% and *P* < 0.1. When no heterogeneity was presented, the results from single comparisons were combined using a fixed-effect model with the Mantel-Haenszel method. Otherwise, the random-effect model with the DerSimonian-Laird method was applied for pooling.

For the reason that the characteristics of target populations, study location, study designs, assessments of smoking, and adjustments for confounding factors were not consistent among studies, we further conducted additional subgroup and sensitivity analyses to explore possible explanations of heterogeneity and to assess the potential impact modification of these variables on outcomes. We also analyzed the effect of a single study on the overall risk estimate by omitting one study in each turn. Due to rather small numbers of studies for other outcomes, the sensitivity analysis was performed only for “ever versus never smoking”. The presence of publication bias will be assessed by applying the tests proposed by Begg’s and Egger’s tests. *P* < 0.05 was considered indicative of significant publication bias. All *P* values were two-sided.

## Results

### Search results and characteristics of studies

**Fig. 1:**
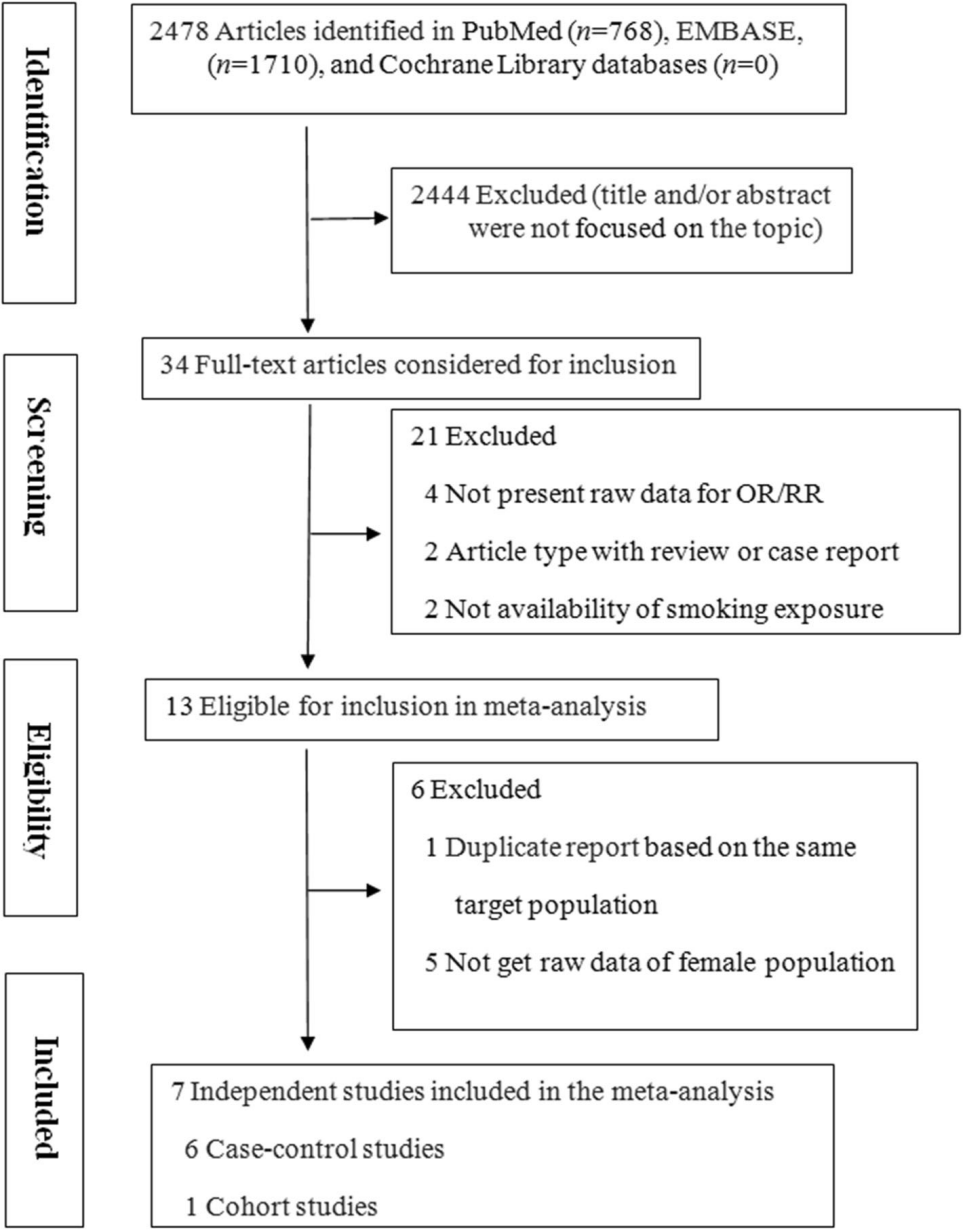
Flowchart of selection of studies for inclusion in meta-analysis

Figure 1 shows a flowchart of the selection process for relevant studies. A total of 2478 (non-unique) publications were first identified in PubMed (*n*=768), EMBASE (*n*=1710), and Cochrane Library databases (*n*=0). Most of these articles were not focused on the topic, and were no longer considered, whereas 34 unique publications were selected for full-text review. A total of 21 articles were excluded because of not present raw data for OR/RR (*n*=4), article type with review or case report (*n*=2), not availability of smoking exposure (*n*=2), and no quantitative estimating of association between smoking and risk of meningioma (*n*=13). Then, 13 publications were retained for the review. One article [23] was excluded because it was one of the multiple reports from the same study [17]. Five articles were excluded because they could not get raw data of female population, and were therefore not comparable to other studies [18, 19, 24–26]. Finally, seven publications were included in the present meta-analysis [12–17, 21].

Table 1 shows the main characteristics of the seven studies included in the present meta-analysis. There were six case-control and one cohort studies. Of these, three studies were conducted in the USA, one in Canada, UK, Israel, and China. Overall, there were 4004 female participants in the case-control studies, of whom 1760 were meningioma cases, while there were 1,177,087 participants in the cohort studies, of whom 372 were meningioma cases.

**Table 1:** Description of epidemiological studies on smoking and meningioma in women included in the meta-analysis

Studies	Country	No. of female cases/controls	Smoking rate (%,F+M/F)	Type of control	Research instrument	Information on smoking	Variables assessed and adjustment
Case-control studies							
Flint-Richter et al. 2011 [15]	Israel	171/196	28.49/23.70	Hospital	Face-to-face interview	Ever/never, pack-years	Adjusted for radiation exposure
Lee et al. 2006 [13]	USA	217/248	NA/17.32	Hospital	Mailed questionnaires	Ever/never, current, past	* Adjusted for age, race, menarche, pregnancy, menopause, oral contraceptives, thyroid disorders, and radiation treatment
Phillips et al. 2005 [16]	USA	143/286	50.50/48.48	Population	Interview	Ever/never, pack-years, duration, age at start, amount	Adjusted for education.
Hu et al. 1999 [12]	China	113/226	35.15/13.86	Hospital	Interview	Ever/never, pack-years, duration, age at start	Adjusted for income, education, occupational exposure to chemicals, and consumption of fruit and vegetables.
Vida et al. 2014 [21]	Canada	67/331	23.15/20.04	Population	Interview	Ever/never, current, pack-years, duration	Adjusted for age, sex, education level, and region
Claus et al. 2012 [17]	USA	1 049/957	10.82/10.61	Hospital	Interview	Ever/never, current, past, duration, pack-years	Adjusted for race, age, BMI, alcohol use, and education
Cohort studies							
Benson et al. 2008 [14]	UK	372/1, 176, 715	NA/20.80	Population	Mailed questionnaires	Never/past, current	* Adjusted for height, body mass index, socioeconomic status, alcohol intake, strenuous exercise, age at first birth, parity, and oral contraceptive use

### Ever versus never smoking

**Fig. 2:**
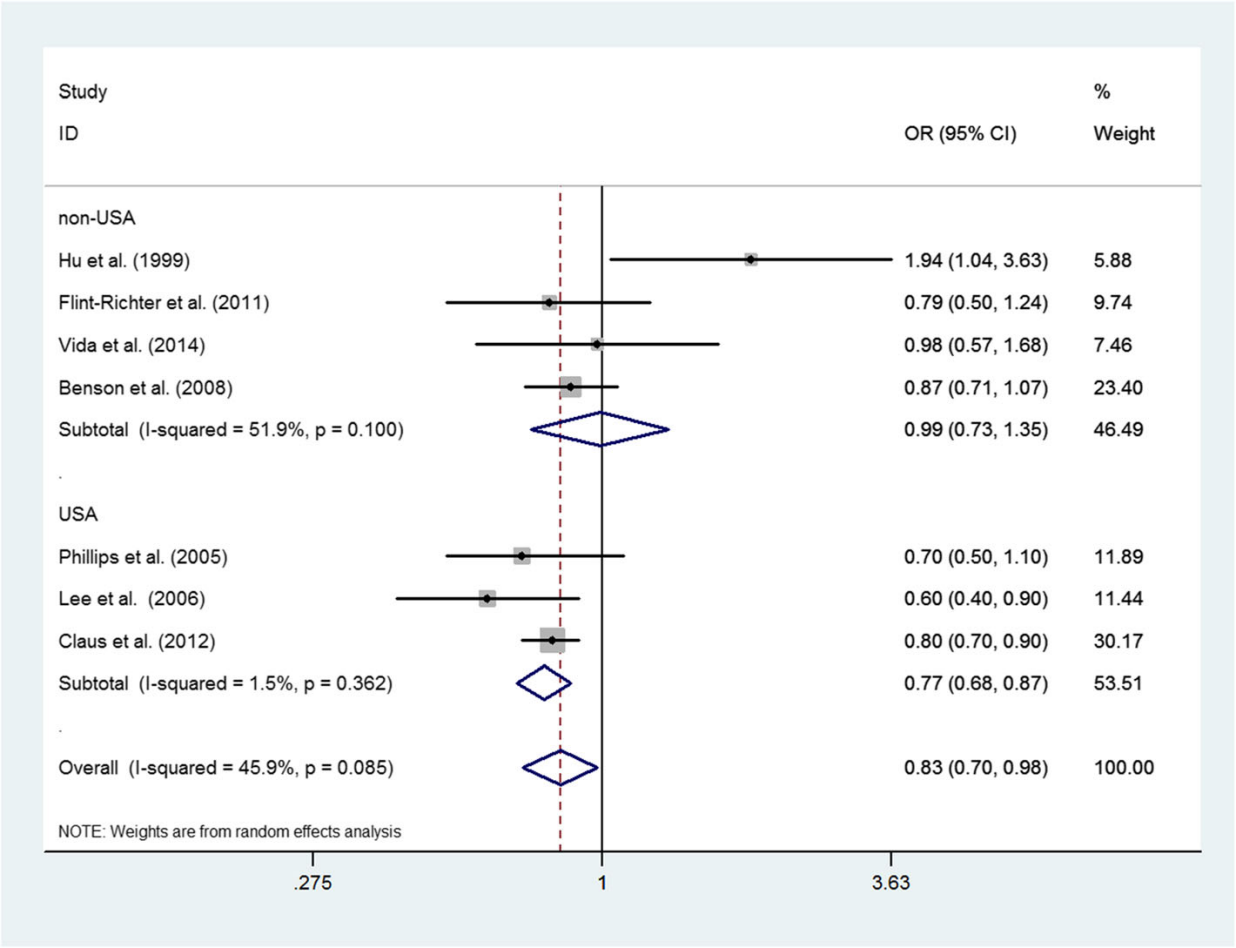
Pooled OR and 95% CI for the association between ever versus never smoking and risk of meningioma in women. Ever smoking was associated with a significantly reduced risk of meningioma in women, with pooled OR of 0.83 (95% CI 0.70–0.98, *P* for heterogeneity, 0.085). Considering the area, the OR of ever smoking was 0.77 (95% CI 0.68–0.87, *P* for heterogeneity, 0.362) in three American studies, 0.99 (95% CI 0.73–1.35, *P* for heterogeneity, 0.100) in four studies conducted in other countries.

Figure 2 shows the study-specific and pooled ORs and 95% CIs of meningiomas for ever versus never smoking in women. A total of seven studies reported the risk of meningioma for ever versus never smoking in women. Three studies were conducted in the USA and four studies were conducted in other countries. Among three studies conducted in the USA, the pooled OR was 0.77 (95% CI 0.68–0.87; *P* for heterogeneity, 0.362). Among four studies conducted in other countries, the pooled OR was 0.99 (95% CI 0.73–1.35; *P* for heterogeneity, 0.100). Overall, the OR was 0.83 (95% CI 0.70–0.98; *P* for heterogeneity, 0.085).

### Never versus current or past smoking

**Fig. 3:**
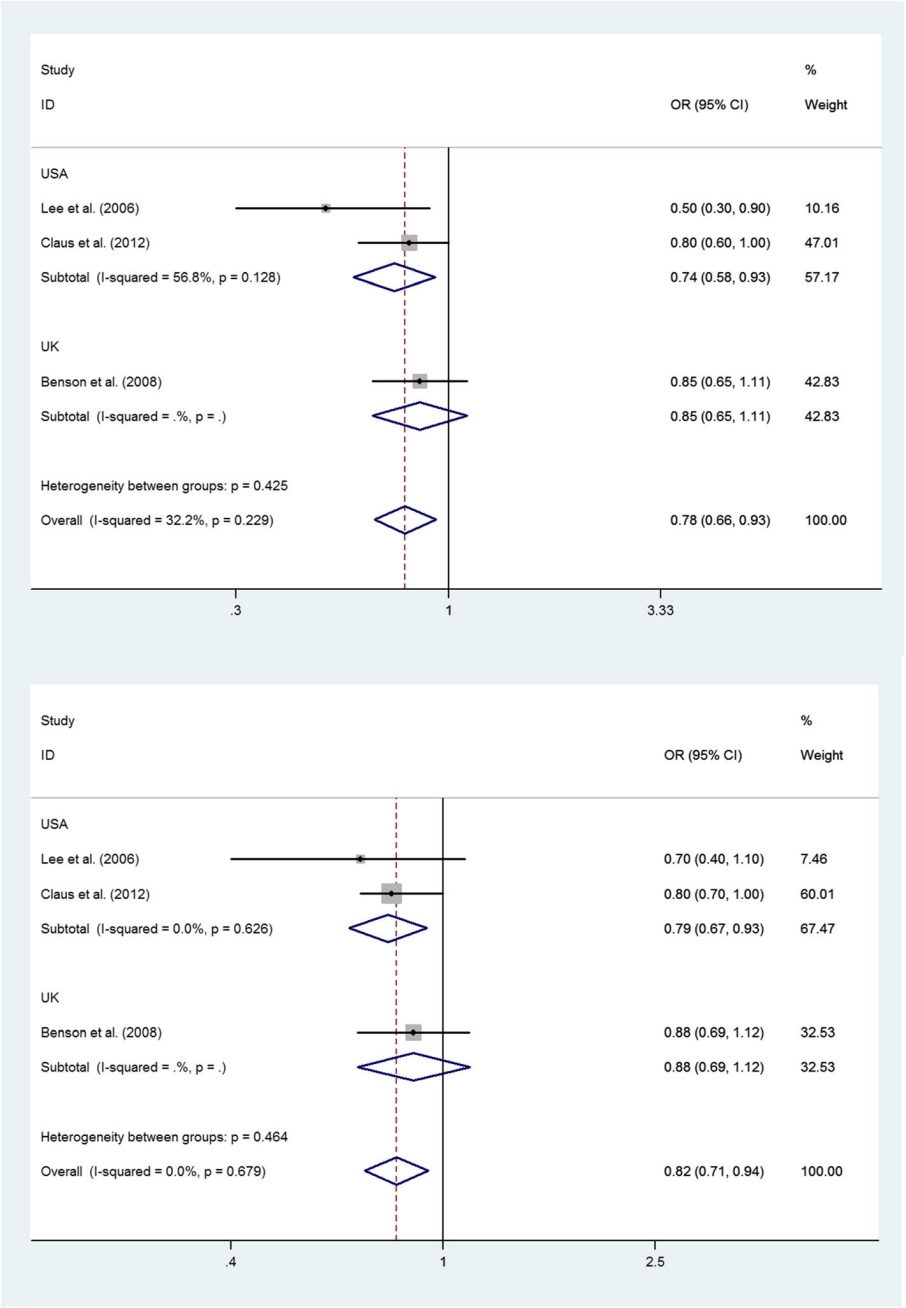
Pooled OR and 95% CI for the association between never versus current or past smoking and risk of meningioma in women. Current smoking was associated with a significantly reduced risk of meningioma in women, with pooled OR of 0.78 (95% CI 0.66–0.93; *P* for heterogeneity, 0.229, **A**). Past smoking was associated with a significantly reduced risk of meningioma in women, with pooled OR of 0.82 (95% CI 0.71–0.94; *P* for heterogeneity, 0.679, **B**)

Figure 3 shows the study-specific and pooled ORs and 95% CIs of meningioma for current and past versus ever smoking in women. The meta-analysis of current (versus never) smoking in women included three studies, with pooled OR of 0.78 (95% CI 0.66–0.93; *P* for heterogeneity, 0.229) for total studies (Fig. 3A). The meta-analysis of past (versus never) smoking in women included three studies, with pooled OR of 0.82 (95% CI 0.71–0.94; *P* for heterogeneity, 0.679) for total studies (Fig. 3B).

### Sensitivity analyses and additional subgroup analyses

Table 2 gives pooled ORs and corresponding 95% CI of meningioma for ever versus never smoking in strata of selected factors. Considering the development level, the OR of ever smoking was 0.80 (95% CI 0.73-0.88) in the developed countries. Considering the area, the OR of ever smoking was 0.77 (95% CI 0.68-0.87) in USA. However, the relationship between ever smoking and meningioma risk was not significantly modified by the target population, study design, smoking assessment. Particularly, when the data were restricted to studies reported adjusted OR, it resulted in a null association between the two. Besides, subgroup analyses indicated that the moderate study heterogeneity was entirely due to a single study (*I*^*2*^= 0% when Hu et al. was dropped from the meta-analysis).

**Table 2:** Pooled ORs and 95% CI for the association between ever versus never smoking and risk of meningioma in women, according to selected subgroups

Subgroups	No. of studies	OR (95% CI)	*I*^*2*^	*P*
Overall	7	0.83 (0.7–0.98)	45.9	0.089
Target population				
Female	2	0.75 (0.53–1.07)	61.1	0.109
Male + female	5	0.88 (0.69–1.13)	53.0	0.075
Area				
USA	3	0.77 (0.68–0.87)	1.5	0.362
Europe and Canada	2	0.88 (0.73–1.07)	0	0.686
Asia	2	1.21 (0.50–2.90)	80.7	0.023
Development level				
Developed country	6	0.80 (0.73–0.88)	0	0.614
Developing country	1	1.94 (1.04–3.62)	-	-
Study design				
Cohort	1	0.87 (0.71–1.07)	-	-
Case control	6	0.83 (0.66–1.04)	53.0	0.059
Hospital-based	4	0.86 (0.62–1.19)	68.9	0.022
Population-based	2	0.79 (0.57–1.08)	0	0.324
Smoking assessment				
Interview	5	0.88 (0.69–1.13)	53.0	0.075
Questionnaires	2	0.75 (0.53–1.07)	61.1	0.109
OR type				
Crude	2	0.75 (0.53–1.07)	61.1	0.109
Adjusted	5	0.88 (0.69–1.13)	53.0	0.075

*OR*, odds ratio; *95% CI*, 95% confidence interval

**Fig. 4:**
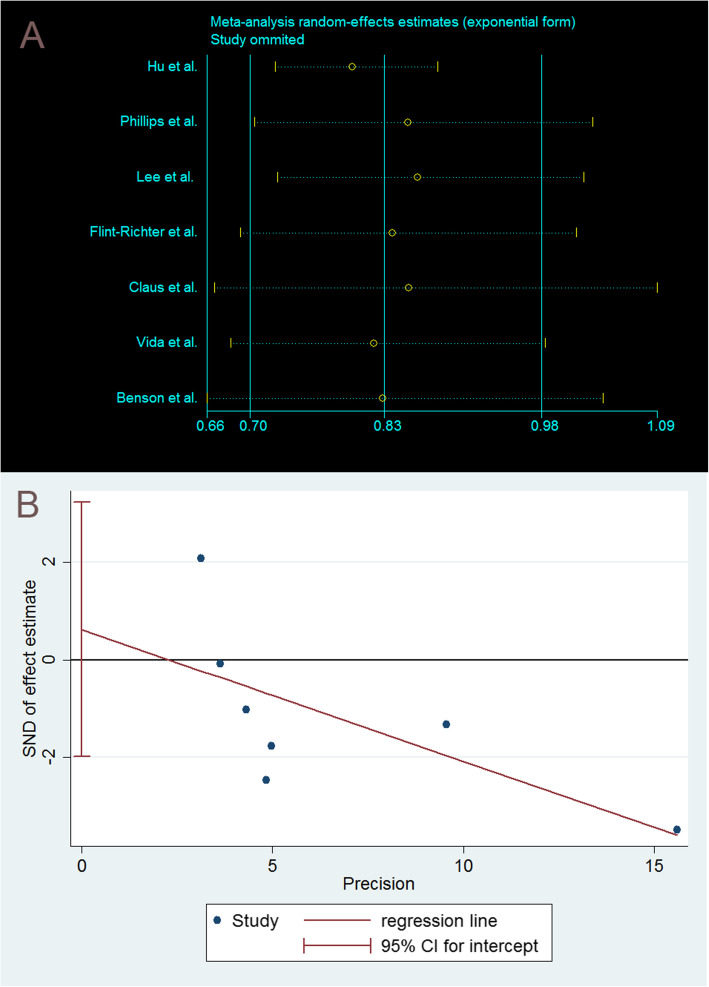
Sensitivity analyses and publication bias assessed by Egger’s tests of seven included studies in this meta-analysis. Exclusion of a single study conducted in China changed the overall risk estimate obviously, with a lower OR of 0.80 (95% CI: 0.73–0.88, **A**). The Egger’s (*P*=0.565) and Begg’s (*P*= 0.368) tests confirmed no evidence of publication bias

Sensitivity analyses showed that the exclusion of five single studies could materially alter the overall combined risk estimate, with a narrow range from 0.83 (95% CI: 0.66–1.04) to 0.86 (95% CI: 0.73–1.02). However, exclusion of a single study [12] conducted in China changed the overall risk estimate obviously, with a lower OR of 0.80 (95% CI: 0.73–0.88) (Fig. 4A).

### Publication bias

Figure 4B shows visually a symmetrical distribution of published studies for the association between ever smoking and meningioma in women. The graph did not show meaningful asymmetry of the studies. The Egger’s (*P*=0.565) and Begg’s (*P*= 0.368) tests confirmed no evidence of publication bias.

## Discussion

In the present meta-analysis, we reviewed and summarized the extensive but controversial evidence on the association between smoking and meningioma in women. We found that ever smoking was associated with a significantly reduced risk of meningioma in women. Similar findings were noted for current and past smokers. Importantly, a decreased risk of meningioma in women smokers was only observed in American studies rather than studies conducted in other countries according to the results of subgroup analyses. However, these findings were based on limited epidemiological evidence, including seven studies with 2132 female meningioma cases that provided risk estimates for smoking.

The possible role of smoking in the development of meningioma in women is still unclear. It is widely recognized that meningioma is a hormone-sensitive tumor at a population level, with approximately 30% of meningioma expressing estrogen receptors and approximately 70% expressing progesterone receptors [27]. An association between hormones and meningioma risk has previously been reported by numerous studies [28, 29]. Moreover, longer exposure to the effect of female sex hormones (such as hormonal replacement therapy) may increase the risk of meningioma in women [30, 31]. It was suggested that smoking has an antiestrogenic effect in women by three potential mechanisms: (1) induction of enzymes that produce low biopotent estrogens, (2) competitive inhibition by binding to estrogen receptor, and (3) decreased activity of aromatases with a subsequent reduction in steroid production [32]. Obviously, our results support the hypothesis that smoking may reduce the risk of meningioma in women by antiestrogenic effect. However, whether smoking has an antiestrogenic effect in female meningioma cases is unexplored. This hypothesis is an intriguing one which could be usefully explored in further research.

Our results are in accord with a previous meta-analysis which indicated that a statistically significant negative association between ever smoking and meningioma in women is found [17]. However, compared with the previous study, the main strength in our study is that we conducted detailed subgroup analyses regarding smoking in relation to meningioma in women [17]. The most interesting finding is that studies conducted in the USA showed a significantly lower risk of meningioma in female smokers [13, 16, 17]. Conversely, no significantly decreased risk of meningioma in female smokers was found in studies conducted in other countries [14 15 21]. It is difficult to explain this result. There is one possible explanation for this result. It was observed that high socioeconomic status (e.g., university education, intermediate non-manual occupation) is related to increased risk of meningioma in women in a population-based cohort study [33]. On the one hand, smoking behaviors may differ across countries with different socioeconomic development levels. For instance, the prevalence of smoking is inversely related to socioeconomic status in most developed countries (developed countries are the countries which are developed in terms of economy and industrialization. The developed countries are also known as Advanced countries or the first world countries, as they are self-sufficient nations.) [34, 35]. On the contrary, in most developing countries (the countries which are going through the initial levels of industrial development along with low per capita income are known as developing countries.), a positive association between socioeconomic status and tobacco use existed, especially for older women [36, 37]. The USA has the highest socioeconomic development level in the world. It is now well established from a variety of studies, that low socioeconomic status is strongly related to an increased smoking prevalence in the USA, with a rate of decline varied by educational level and occupational class [38-40]. On the other hand, cigarette smoking behaviors may also differ across races. The main race of the target population in American study is Caucasian, but the main race of the target population in other studies is multi-racial including Asian and Israel. Meanwhile, a recent study found that low socioeconomic status is generally associated with increased cigarette smoking prevalence in Caucasian women, but this result is not observed in Asian women [38]. It means that American female smokers may have a greater proportion of individuals with low socioeconomic status than Asian female smokers. Therefore, American female smokers with low socioeconomic status may have a lower risk of meningioma. If these socioeconomic factors are not well controlled, the cross interactions between socioeconomic status and smoking may have a potential negative impact on the risk of meningioma for smoking in American studies. In spite of that, considering socioeconomic factors, two American studies have only adjusted for education, while another one did not adjust for any socioeconomic factors [13, 16, 17]. In contrast, Hu et al. have adjusted not only for education but also for income in their study [12]. Hence, it could conceivably be hypothesized that the negative cross interactions between socioeconomic status and smoking may underestimate the risk of meningioma for smoking in American studies. Further studies, which take these variables into account, will need to be undertaken. Another strength of this study is the possibility to examine the relation with meningioma in women separately for the ever smoking subtypes, i.e., past and current. Our meta-analysis is novel in that past or current smoking status is also associated with a significantly reduced risk of meningioma in women, although these findings are based on only three studies. Furthermore, the third strength is that our meta-analysis is based on the updated epidemiological data, and included more relevant studies than previous meta-analyses.

Despite the intriguing findings of our study, several important limitations should be considered. Firstly, the major limitation of this study is the small number of studies: only seven studies were included in our meta-analysis of meningioma risk associated with ever versus never smoking. Meanwhile, only three studies were included to analyze the association between past or current smoking and risk of meningioma in women. Secondly, meningiomas are rare and most patients are asymptomatic, making it difficult to get high-quality information in epidemiological research. In our study, only one prospective cohort study was included, and most of the information on this topic came from case-control studies, which may be affected by various sources of bias. Furthermore, as the current studies use different assessment instruments (interview or mailed questionnaires) to assess the participants’ smoking status, the possibility of mis-reporting of cigarette smoking by study participants may exist. With different methods, the study participants may have different attitudes, which could affect the accuracy of the data collection. However, subgroup analyses based on the different study design and the different assessment instruments got consistent results. Thirdly, considering the multivariate adjusted OR values reported in the literature are the most precise risk estimates, these values were used in our meta-analysis preferably. However, not all included studies in this meta-analysis reported the multivariate adjusted OR. In fact, the variables of adjustment in current studies are inconsistent. Unfortunately, when the data were restricted to studies that reported adjusted OR, it resulted in a null association between the two. Therefore, evidence for an apparent protective effect of ever smoking on risk for meningioma in women is still weak.

## Conclusions

In conclusion, based on limited epidemiological evidence, ever smoking (including past or current smoking) is related to a decreased risk of meningioma in women. Importantly, a decreased risk of meningioma in women smokers was only observed in American studies rather than studies conducted in other countries according to the results of subgroup analyses. Thus, we infer that smoking behaviors differ across socioeconomic development levels and race may be the explanations of the inconsistent results in different studies. However, the evidence for this protective effect of smoking on risk for meningioma in women is still weak, particularly when the data are restricted to studies reported adjusted OR, which resulted in a null association between the two. Therefore, there remains a need for further prospective cohort studies including adequate numbers of cases that can more clearly evaluate the temporal relationship between smoking and risk of meningioma in women.

## Data Availability

Please contact the author for data requests.
